# Wear of Titanium Implant Platforms with Different Abutment Connections and Abutment Materials: A Pilot Study

**DOI:** 10.3390/jfb14040178

**Published:** 2023-03-23

**Authors:** Teresa A. Mendes, Luis Vilhena, Jaime Portugal, João Caramês, Amilcar L. Ramalho, Luis P. Lopes

**Affiliations:** 1 Faculdade de Medicina Dentária, Universidade de Lisboa, 1600-277 Lisbon, Portugal; 2 Department of Mechanical Engineering, Centre for Mechanical Engineering, Materials and Processes (CEMMPRE), University of Coimbra, 3004-516 Coimbra, Portugal; 3 Laboratório de Instrumentação, Engenharia Biomédica e Física da Radiação (LIBPhys), 1600-277 Lisbon, Portugal

**Keywords:** implant, wear, abutment, connection, titanium, zirconia

## Abstract

The most commonly used material in dental implants and their abutments is titanium. Zirconia is a more aesthetic alternative to titanium abutments; however, it is much harder. There are concerns that zirconia could damage the surface of the implant over time, especially in less stable connections. The aim was to evaluate the wear of implants with different platforms connected to titanium and zirconia abutments. A total of six implants were evaluated, two of each connection type: external hexagon, tri-channel, and conical connections (n = 2). Half of the implants were connected to zirconia abutments, and the other half to titanium abutments (n = 3). The implants were then cyclically loaded. The implant platforms were evaluated by digital superimposing micro CT files and calculating the area of the loss surface (wear). In all the implants, a statistically significant loss of the surface area (*p* = 0.028) was observed when comparing the area before and after cyclic loading. The average lost surface area was 0.38 mm^2^ with titanium abutments and 0.41 mm^2^ with zirconia abutments. The average lost surface area was 0.41 mm^2^ with the external hexagon, 0.38 mm^2^ with the tri-channel, and 0.40 mm^2^ with the conical connection. In conclusion, the cyclic loads induced implant wear. However, neither the type of abutment (*p* = 0.700) nor the connection (*p* = 0.718) influenced the amount of surface area lost.

## 1. Introduction

Dental implants allow the replacement of missing teeth [1]. The most commonly and traditionally used material to produce dental implants is grade 4 titanium [2]. This is a biocompatible material with a low risk of corrosion that is ideal for endosseous applications [3].

The implants are rehabilitated with prosthetic crowns with the abutments being responsible for the connection between the implant and the crown. The most commonly used material to manufacture abutments is grade 5 titanium alloy. In this alloy, other elements such as aluminum and vanadium are added to increase the mechanical strength [2]. However, there are concerns about the toxicity of these added elements [4,5]. Titanium is gray in color, which can compromise the aesthetic of soft tissues [6,7], especially in thinner soft tissue biotypes [8].

To overcome these limitations, the first zirconia abutments were introduced in 1997 [9]. These abutments have high mechanical resistance, biocompatibility, and better aesthetic properties compared with titanium [10]. Furthermore, they are harder (1300 HV-Vickers Hardness Scale) than grade 4 dental titanium implants (263 HV) and grade 5 titanium abutments (320 HV) [11], and there are concerns that zirconia abutments screwed directly to the implant will cause wear on the titanium surface during function, thus leading to a loss of implant platform geometry and instability in the implant–abutment connection [12,13].

Other solutions have been studied, such as the use of titanium abutment with CrN/NbN coating. This material can influence the peri-implant tissue behavior compared with the traditional machined abutment [14]. Tissue-level implants seem to be a valid alternative to traditional implants, with and without abutments. New tissue-level implant designs with convergent collars may be used without implant abutment units. Simplifying the prosthetic procedure by not using abutments may result in a decrease in costs, the prevention of microgaps at the bone level, and facilitation of the emergent profiles. A microgap that is distant from the bone level may reduce both the bacterial contamination and peri-implant mucosa trauma that may occur during unscrewing and screwing prosthetic procedures [15].

The implant–abutment connection is generally described as an external or internal connection according to its geometry [16]. The external hexagon is a type of external connection with a hexagon with a height of 0.7 mm, which extends above the coronal surface of the implant platform. This connection was introduced by Branemark and is still used today with the advantage of having multiple prosthetic solutions [17]. Its disadvantages are the low height of the hexagon, which reduces the abutment–implant contact area [18], creating stress in the prosthetic connection screw [16,18]. This connection is associated with an increased incidence of screw loosening [19]. There are several types of internal connections according to the geometry of the indexing features, which are the internal hexagon, tri-channel, conical connection, and cone morse [20]. In the internal connections, the geometric features are extended into the implant, increasing the implant–abutment contact area and the stability of their joint [21]. This connection has a lower incidence of screw loosening and a better joint strength [19,22]. One of the first internal connections, the Core Vent^®^, was developed by Niznick in 1983. It is composed of an internal hexagon with a depth of 1.7 mm [23]. Many different internal hexagon configurations are currently available [16]. Those that have an associated cone offer better resistance to lateral movements [24]. The tri-channel connection is characterized by the existence of three lateral channels on the internal surface of the implant. The long tube of this connection creates good lateral stability, while the side channels allow for precise indexing [16]. The morse taper connection is a specific type of conical connection. The original morse taper concept is characterized by the existence of an abutment with a specific angulation of 1 to 2°, which adapts to an identical surface on the implant without any thread system [16]. 

Previous authors have reported greater wear when implants were screwed to zirconia abutments than when they were screwed to titanium abutments [12,25,26]. However, these authors did not measure the wear as the total area of loss of implant platform but as arbitrary linear points [12,25] or indirectly by dark spots on the abutments [26]. Additionally, they did not simulate the oral cavity conditions, for example, the presence of saliva, which can act as a lubricant [27]. To our knowledge, no studies have been able to quantify the wear of titanium implants with different connections and abutments.

The purpose of this study was to analyze and measure the wear of grade 4 titanium implants with different platforms connected to grade 5 titanium or zirconia abutments after cyclic loading. 

The following null hypotheses were tested: (1) the cyclical load does not affect the lost surface area, (2) the abutment material (titanium or zirconia) does not affect the lost surface area, and (3) the connection type (external hexagon, tri-channel, or conical connection) does not affect the lost surface area.

## 2. Materials and Methods

A total of six implants were evaluated, two implants of each type of connection: external hexagon (Branemark MK III TiUnite RP Nobel Biocare, Göteborg, Sweden), tri-channel (Replace Tapered RP Nobel Biocare, Göteborg, Sweden), and conical (Nobel Active RP Nobel Biocare, Göteborg, Sweden), n = 2. Each implant was inserted in the center of an epoxy resin block (DPC-Laminierharz LT 2, Duroplast-ChemieVertriebs GmbH, Neustadt/Wied, Germany) in accordance with the respective manufacture chirurgical protocols with the implant platform 3 mm above the resin level. Half of the implants were connected to zirconia abutments (Zirkon Translucence, Zirkonzahn GmbH, Gais, Italy) and the other half were connected to grade 5 titanium abutments (Titan5 Zirkonzahn GmbH, Gais, Italy), forming 6 different implant–abutment combinations. Both the titanium and zirconia abutments were designed using Zirkonzhan software (ZirkonzhanModelier, Gais, Italy) and produced with CAD-CAM (Computer-Aided Design—Computer-Aided Manufacturing) techniques (Zirkonzahn M5, Gais, Italy). They had a width of 5 mm, a height of 8 mm, and a 30° incisal edge inclination [28]. Each abutment was screwed to the respective implant using a suitable screw for zirconia or titanium abutments (Abutment screw, Zirkonzhan, Gais, Italy) and applying a torque of 35 Ncm with a manual torque wrench (Nobel Biocare, Göteborg, Sweden).

**Figure 1:**
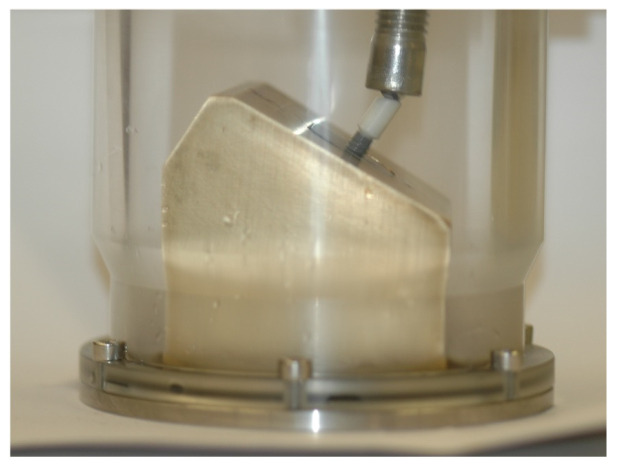
Testing machine with specimen support.

The samples were submersed in artificial saliva SAGF [29] and submitted to 1,200,000 load cycles with a sinusoidal load ranging between 10 N and 100 N [11,27] at a frequency of 10 Hz [28] in a fatigue testing machine (Instron Electro Plus E10000, Instron, Norwood, MA, USA). The cyclic load was applied with an angulation of 30º to the long axis of the implant (Figure 1) [28].

For each implant, two micro CTs (Micrograph Computer Tomograph) were performed, one before abutment placement and load application in order to obtain an unloaded implant image and the second after the application of the load and the removal of the abutment to obtain the image after load application. The equipment (Metrotom 800, Zeiss GmbH, Oberkochen, Germany) had 130 kV and an acceleration of 300 μA with a voxel size of 7.86 μm. The reading was carried out by rotating 360° around the implant, and images were taken every 0.36°. The data obtained were exported to an STL file (Surface Tessellation Language) from the micro-CT software (Metrotom OS, Oberkochen, Germany). The STL files were imported to the Geomagic Control X64 software version 2021.0.3. In this software, the STL file collected prior to cyclic loading for each implant was superimposed onto the STL file of the same implant after cyclic loading with toll best-fit alignment. The pattern of three-dimensional deviations between the two models was represented by a respective color scale. The negative deviations were represented on a scale of blue and purple, corresponding to the wear zones after load application [25,30].

The digital superimposed files of each implant were longitudinally sectioned along the zone with major alterations. With the 2D (two-dimensional) toll, the linear deviations in that zone were measured to obtain the linear wear values. 

**Figure 2:**
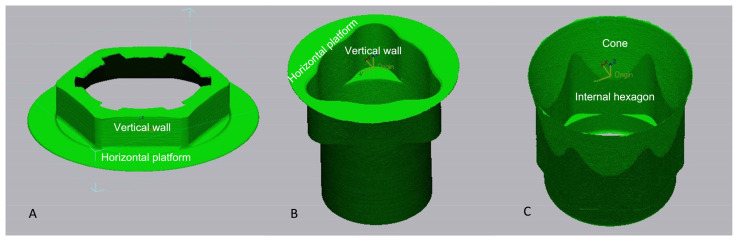
Area of the platform accounting for the external connection (**A**), “tri-channel” (**B**), and conical connection (**C**).

The implant was segmented into multiple geometric components with the toll auto segments (Geomagic ControlX64 software). The lost surface area was calculated from the sum of the components of the zones of the implant–abutment connection before and after cyclic loading. The summed component for each connection and each component name is illustrated in Figure 2.

The implant platform at the indexing zones was observed with a scanning electron microscope (SEM) (Jeol, Jsm7001F, Tokyo, Japan) after cyclic loading with 22× and 1000× amplifications.

To measure the repeatability between different readings performed by the micro CT device, an initial reliability test was performed [31]. Two different readings were obtained from the same implant before applying the load (R1 and R2). The variation of the surface area of the platform between the two readings was recorded. Micro CT techniques have validity and produce similar results to traditional destructive techniques with physical measures [32].

Since all samples were measured by the same operator, calibration was performed during data collection to determine whether there was consistency in reading the results. To assess the intraobserver variability, the measurements were repeated 4 weeks after the first session. No identifiers were present on the images to minimize bias [33].

Data were statistically analyzed using SPSS Statistics, version 28.0.1 (IBM SPSS Statistics, New York, NY, USA). 

Although normality was verified with Shapiro–Wilk test (*p* > 0.05), non-parametric tests were used due to small sample size. A confidence level of α = 0.05 was assumed for all statistical tests.

The intraobserver variability was assessed using Wilcoxon tests between the first and second measurement sessions. 

The dependent variable was the lost surface area (continuous variable, values expressed in mm^2^), and the independent variables were the abutment type and the connection type. The values of the surface area before and after the application of loads were compared with the Wilcoxon nonparametric tests. The difference between the initial and final area was also compared according to the type of abutment and connection.

The data obtained were grouped according to the different types of abutments and analyzed using the Mann–Whitney U test. The data were also grouped according to the type of connection and analyzed using the Kruskal–Wallis tests. The values of linear deviations in the implant platforms were not submitted to statistical analysis because the points of measurement were randomly chosen. 

## 3. Results

### 3.1. Reliability Test and Intraoperator Calibration

The readings of the platform surface area were 29.1341 mm^2^ and 29.0989 mm^2^ (R1 and R2). A difference of 0.0352 mm^2^ was recorded between the two readings for the same implant. The average difference between readings in session one and session two (after 4 weeks) were 29.41 and 29.40 mm^2^, respectively. The Wilcoxon test revealed there was no significant difference between measurements performed on the same implants initially and after 4 weeks (*p* = 0.655). 

### 3.2. Digital Superimposing

**Figure 3:**
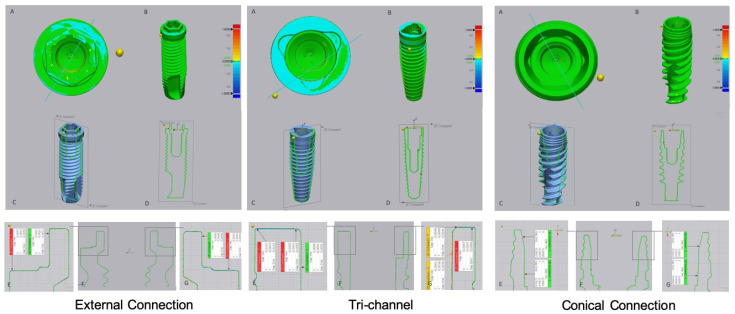
Images of digital superimposed micro CT files of titanium implants with external hexagon, tri-channel, and conical connection platforms after having been connected to titanium abutments and submitted to cyclic loading. (**A**)—occlusal view; (**B**)—frontal view; (**C**,**D**)—schematic implant section; (**E**–**G**)—linear deviation measurements of the platform from the buccal to palatine areas.

**Figure 4:**
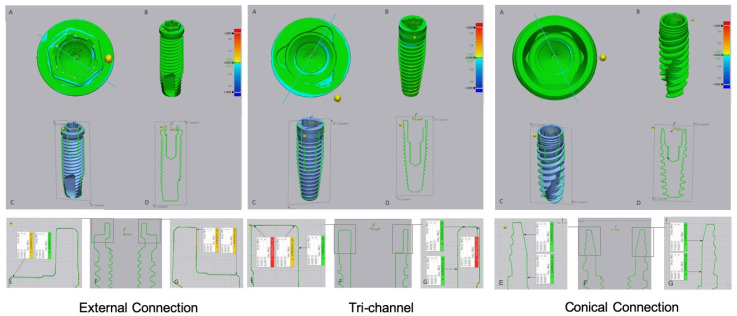
Images of digital superimposed micro CT files of titanium implants with external hexagon, tri-channel, and conical connection platforms after having been connected to zirconia abutments and submitted to cyclic loading. (**A**)—occlusal view; (**B**)—frontal view; (**C**,**D**)—schematic implant section; (**E**–**G**)—linear deviation measurements of the platform from the buccal to palatine areas.

The results of the digital superimposition and digital section of the files before and after cyclical loading are presented in Figure 3 (titanium abutment) and Figure 4 (zirconia abutment). In the external connection, an extended blue zone was observed on the implant platform that was connected to the titanium abutment (Figure 3, external connection A). The blue colored zones were located on the horizontal base of the platform and the edges of the hexagon, although they were not evident on the vertical walls and vertices.

The tri-channel connection also showed wear zones with greater extension in the implant that was connected with the titanium abutment. These zones were most evident on the periphery of the horizontal platform in relation to the interior part of the connection (Figure 3, tri channel A, E, G). In the tri-channel connection, an almost perfect ring was observed with the implant that was connected to the zirconia abutment (Figure 4, tri channel A).

In the conical connection, blue zones were not observed with zirconia or titanium abutments (Figure 3 and Figure 4, conical connection A).

### 3.3. Linear Deviation Measures

By analyzing the values presented in Table 1, it can be observed that, for the external hexagon and tri-channel connections, the linear deviation values were higher on the horizontal platform compared to those verified in the vertical walls. In the tri-channel connection, the values were higher at the periphery of the platform and decreased as the internal limit approached.

In the conical connection, it can be observed that the wear was distributed along the cone, being less evident in the internal hexagon.

### 3.4. Lost Surface Area

The values related to the surface area of the implant platform before and after the application of the cyclic loads, as well as the difference recorded, are shown in Table 2, Table 3, Table 4 and Table 5.

The application of cyclic loads led to a statistically significant reduction (*p* = 0.028) in the surface area of the implant platform. However, the wear was not influenced by the type of abutment (*p* = 0.700) or the type of connection (*p* = 0.718).

### 3.5. SEM Images

**Figure 5:**
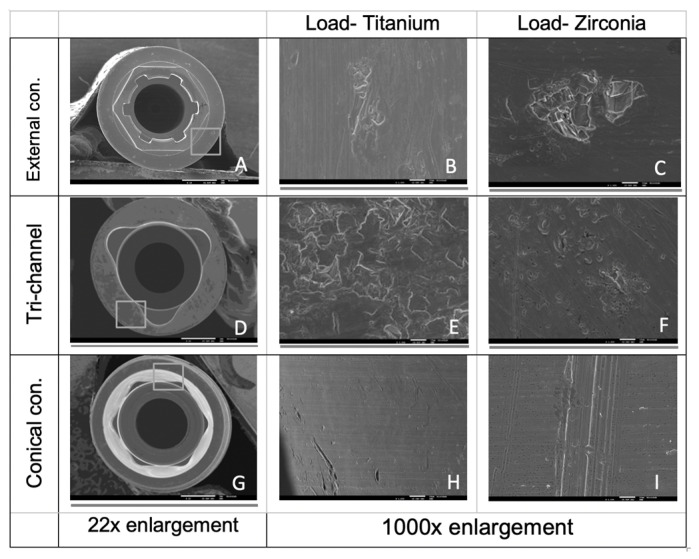
SEM images of the horizontal platform of implants with external and tri-channel connections (**A**–**F**) and of the cone area in conical connection implants (**G**–**I**).

SEM images after cyclic loading of the implants are presented in Figure 5. In all the connections, taking into account the images obtained in the areas corresponding to wear in the digitally superimposed micro CT files, scratches and abrasions were detected on images with higher magnification levels. 

## 4. Discussion

This preliminary study demonstrated that the test method produced quantitative data to measure the surface area lost from the implant platform before and after cyclical loading using Geomagic software with micro CT digital analysis. The surface area lost from the implant platform was considered to represent implant wear. It was also possible to observe the tri-dimensional wear pattern and to measure it through the digital section linear deviations between the measurements made before and after loading. The experimental model used was intended to simulate clinical conditions and was developed according to the ISO 14801-2007 standard. 

To ensure the repeatability of the method used, an initial reliability test was performed. The difference between the two readings was 0.03 mm^2^. Previous studies with larger sample sizes have demonstrated that there is intra micro CT system reproducibility [34]. We consider the micro CT device used to be adequate for the purposes of this study. The intraobserver variation between readings was lower. 

The wear pattern in the external hexagon and tri-channel connections was similar with more wear and linear deviations in the horizontal base of the platforms, as demonstrated by the linear wear values and SEM images. This pattern can be explained by the contact made between the implant abutment, tight on the horizontal platform and with some relief in the external and internal vertical walls [35].

The linear deviation values recorded in the tri-channel connection were higher on the periphery of the implant platform and lower closer to the center of the implant. This can be explained by the fact that when applying loads, the first abutment–implant contact occurs at the periphery of the connection [36].

The wear zones were larger when the load was applied with the titanium abutment in relation to the zirconia abutment with slight differences in the external hexagon and tri-channel connections. This can be explained by the wear mechanism involved: adhesive in the titanium–titanium contact and abrasive in the titanium–zirconia contact [37]. Adhesive wear occurs when two bodies slide over each other, which enhances material transfer between two surfaces with similar physicochemical properties [37]. The abrasive wear is related to the small grain size of zirconia, which creates a less abrasive smoother surface [37]. Some authors used simplified geometry sphere-plane studies in order to simulate the implant–abutment systems. Some studies detected a superior [38,39] or similar wear [40] with the titanium–titanium contact in relation to the titanium–zirconia contact.

The conical connection presented linear deviations on the digitally sectioned implants throughout the entire cone. In this type of connection, it appears that the wear on the implant connected to the zirconia abutment is greater compared to when it is connected to the titanium abutment. The SEM images confirmed these results with visible scratches in the cone zone of the implant loaded with the zirconia abutment.

The results of the present study show a statistically significant (first null hypothesis rejected) reduction in the platform’s surface area after cyclic loading in all implants. This reduction was subsequently evaluated according to the type of abutment material and the type of connection present. In terms of the type of abutment material used, the average value was higher with zirconia abutments than with titanium abutments. However, these differences were not statistically significant (second null hypothesis cannot be rejected). The fact that the statistical analysis was performed with a reduced number of specimens may have contributed to this result. Further studies should use a bigger sample size.

Only a few studies have assessed the wear of implants connected to titanium or zirconia abutments submitted to cyclic loading. These studies showed superior wear with zirconia abutments compared with titanium abutments [12,26,41]. However, the wear assessment methodologies used by these authors differed from those used in the present study. Klotz et al. measured the wear area of the implants indirectly by measuring dark spots on the abutments with electron microscopy. They considered these zones to be transfer regions of titanium from the implant to the abutments [26]. Stimmelmayr et al. calculated the wear of the implant platform with the tri-channel connection at specific points. They performed micro CT readings before and after 1,200,000 load cycles [12]. Subsequently, linear wear points were measured on the implant platforms. Nam et al. digitalized the platform of a conical connection implant with a high precision scanner before and after 100,000 loading cycles. The use of the scanner requires the application of powder on the platform surface of the implant before digitalization. However, this application of powder creates an additional volume on the surface of the implant which can influence the results [25].

Queiroz et al. compared the wear of titanium implants before and after they were cyclically loaded with two different types of abutment: zirconia and a nickel–chromium–titanium alloy. The wear of the external hexagon was analyzed by scanning electron microscopy. The authors found that the zirconia abutments caused more wear on the implants [41].

The divergent results obtained by the different studies can be explained by the distinct methodologies applied (number of cycles, force, and frequency). The advantage of using micro CT is that this technique allows 3D (three-dimensional) files of the total body of the implant to be obtained, and these can be digitally sectioned to measure the wear of all platforms. In the present study, the specimens were also submerged in artificial saliva throughout the test, while in other published studies that evaluated the wear of implants, cyclic loads were applied in an ambient atmosphere or in a saline solution. It remains unclear how wear is influenced by the presence of artificial saliva. Thus, caution is advised when comparing the results of the present study with those of other studies that evaluated implant wear, as the test environment is not the same and its influence on the results is unknown.

Different connections with different areas and geometries have different micromotions at the implant–abutment interface [42]. Low amplitude oscillatory movements can cause implant wear. The external hexagon connection is a less rigid and stable connection than the internal connections [43], which could lead to a different degree of wear. However, in the present study, the average wear values were higher in the external hexagon and with zirconia abutments, but no statistically significant differences were found (third null hypothesis cannot be rejected). Further studies with bigger sample sizes are needed to deepen the knowledge of these phenomena.

This study aimed to investigate the safety of using zirconia abutments directly screwed to implants with different connections. The results of this study indicate that zirconia abutments, although harder than titanium, do not significantly damage the surface of the implants to which they are connected.

Zirconia abutments have aesthetic advantages over titanium abutments, which are gray in color. Titanium abutments require the application of ceramics or adhesion to an aesthetic zirconia crown. Zirconia abutments can also be directly screwed onto the implant, creating a single one-piece abutment [9]. The results of this preliminary indicate that zirconia is a promising material for use in titanium dental implant rehabilitation, but more studies are needed to validate this clinical option.

The main limitation of the present study was the cost of the analyses and the implants used. A very accurate micro CT device with high acceleration was selected. This equipment allows clear images and adequate accuracy tests to be obtained; however, the cost of each analysis is high.

Other limitations of the study are the long test duration, the high number of cycles used, and the fact that few studies have used digital tools to compare implant wear and the methodologies (force, number of cycles, frequency, artificial saliva) employed have been variable [12,24,25]. The ISO standard does not define the magnitude of the force to be applied or the medium used for submerging specimens during load application. 

A clinical trial with real oral conditions is also important to deepen the knowledge of this phenomena.

## 5. Conclusions

The applied model simulated a clinical dental implant system. The digital methodology created allowed us to quantify the wear of dental implants and observe the pattern of wear with different abutment materials and implant connections.

The application of cyclic loads at the abutment level induces wear on the different platforms of titanium implants, but no significant differences were observed between the three connections or between the two types of abutment tested.

In the external hexagon and tri-channel connections, there was more wear on the periphery of the platform base, while in the conical connection, the wear was evenly distributed throughout the cone.

## Figures and Tables

**Table 1:** Values of linear deviations observed in the implant platforms after cyclic loading, according to the experimental groups (values in mm).

Connection	Titanium Abutment				Zirconia Abutment			
	Palatine	Buccal	Palatine	Buccal	Palatine	Buccal	Palatine	Buccal
	Horizontal Platform		Vertical Wall		Horizontal Platform		Vertical Wall	
External Hexagon	−0.0054	−0.0058	−0.0006	−0.0021	−0.0044	−0.0049	−0.0029	−0.0014
Tri-Channel	−0.0068	−0.0102	−0.0038	−0.0001	−0.0070	−0.0079	−0.0008	−0.0004
	Cone		Internal Hexagon		Cone		Internal Hexagon	
Cone connection	−0.0016	−0.0007	−0.0006	−0.0014	−0.0016	−0.0031	−0.0004	−0.0008

**Table 2:** Surface area registered in the implant platforms before and after cyclic loading in different experimental groups (values in mm^2^).

Connection Type	Abutment Type	Area before Loading	Area after Loading	Difference
External hexagon	Titanium	14.39	13.96	0.43
	Zirconia	14.43	14.04	0.39
Tri-channel	Titanium	44.86	44.47	0.39
	Zirconia	44.55	44.18	0.37
Conical connection	Titanium	29.10	28.77	0.33
	Zirconia	29.13	28.67	0.47

**Table 3:** Measures of central tendency of the initial and final areas (mm^2^), n = 6.

	Average (Standard Deviation)	Median (Interquartile Range)
Area before loading	29.41 (13.551)	29.12 (30.209)
Area after loading	29.01 (13.565)	28.72 (30.237)

**Table 4:** Measures of central tendency of the lost surface area, according to the type of abutment (mm^2^), n = 3.

Abutment Type	Average (Standard Deviation)	Median
Titanium	0.38 (0.054)	0.39
Zirconia	0.41 (0.052)	0.39

**Table 5:** Measures of central tendency of the lost surface area, according to the type of connection (mm^2^), n = 2.

Connection Type	Average (Standard Deviation)	Median
External hexagon	0.41 (0.029)	0.41
Tri-channel	0.38 (0.016)	0.38
Conical connection	0.40 (0.100)	0.40

## Data Availability

The research data are available at http://hdl.handle.net/10451/48498 (accessed on 30 September 2020).
